# Characteristics of Mulberry Leaf Powder Enriched With γ-Aminobutyric Acid and Its Antioxidant Capacity as a Potential Functional Food Ingredient

**DOI:** 10.3389/fnut.2022.900718

**Published:** 2022-05-18

**Authors:** Yingchun Jin, Jie Tu, Xinyao Han, Jun Zhuo, Guanhui Liu, Yanhui Han, Hengjun Du, Jun Wang, Hang Xiao

**Affiliations:** ^1^College of Biotechnology, Jiangsu University of Science and Technology, Zhenjiang, China; ^2^Jiangsu Key Laboratory of Sericulture Biology and Biotechnology, Zhenjiang, China; ^3^School of Grain Science and Technology, Jiangsu University of Science and Technology, Zhenjiang, China; ^4^Department of Food Science, University of Massachusetts, Amherst, MA, United States

**Keywords:** AGEs inhibition, DPPH radical scavenging, γ-aminobutyric acid, mulberry leaf, phenolics

## Abstract

To improve the functional properties of mulberry leaves, γ-aminobutyric acid (GABA) enrichment treatments were applied. The results showed that the combined treatment of sodium glutamate immersion, cold shock, and anoxic significantly increased the GABA content. HPLC analysis displayed that the quantity of some active phenolics was significantly increased after the treatment. The GABA-enriched mulberry leaf powders were subsequently prepared, and it was found that as the particle size decreased, their water and oil holding capacity and their swelling power decreased, while the angle of repose increased. The dissolution rate of GABA and total phenolics increased as the particle size decreased. Optical observations and SEM results revealed that the fiber structures of the particles were gradually destroyed as the particle size decreased. Further, FTIR analysis showed that the active compounds in the powders were not destroyed. M400 and M140 powder showed the maximum DPPH radical scavenging ability and AGEs inhibition capacity, respectively. Additionally, adding the powders effectively alleviated the staling of bread without any significant effect on taste.

## Introduction

Mulberry (*Morus alba L.*) is widely cultivated in many countries, and China is the country that preserves the most mulberry germplasm resources (1). It has been reported that various parts of mulberry plants have potential nutritional and pharmacological properties in areas such as anti-oxidation, hypoglycemia, anti-cancer, and anti-inflammation, especially the mulberry leaves (2–4). The health effects of mulberry were first recorded in the Chinese medical book “Shen Nong’s Materia Medica,” which is the earliest known work on Chinese traditional medicine. Modern chemical analysis reveals that mulberry leaves contain a variety of pharmacologically active components including γ-aminobutyric acid (GABA), polyphenols, alkaloids, etc. (5). GABA was reported to have multiple biological neuroprotective, anti-hypertensive, anti-oxidant, anti-anxiety, and anti-inflammatory effects (6). Normally, GABA can be biosynthesized by glutamate decarboxylase in the human body, but the synthesis and accumulation level depends on the age and physical state of the individual (7). So, supplementing GABA in food for the elderly and/or stressed population is a reasonable alternative. The content of GABA in natural products was found to be very low in general, but the average content of GABA in mulberry leaves could reach 4.4–59.4 mg/100 g dry matter (8), which indicates that mulberry leaf is a potential raw material for preparing foods rich in GABA.

Mulberry leaves are rich in glutamate and glutamate decarboxylase, and glutamate can be converted to GABA under certain stress conditions. In addition, it was also found that the phenolic components in mulberry leaves changed significantly during the GABA accumulation. Phenolic components are considered to be functional components of the mulberry leaf with a hypoglycemic effect (9). The biosynthesis of phenolics in the plant was reported as a complex pathway that was affected by a number of different endogenous or exogenous factors, such as salinity, temperature, mechanical damage, and water (10). Ma et al. found that exogenous GABA induced the accumulation of phenolic compounds in germinated hulless barley under NaCl stress and further revealed that GABA was essential for mediating NaCl stress-induced phenolic compound accumulation (11).

The powder is an important form of mulberry leaf development as a food raw material. Hu et al. reported that as the particle size of green tea powder decreased, the content of water-soluble carbohydrates and the extraction rate of tea polysaccharides increased (12). Lin et al. reported that bran micronization increased its soluble dietary fiber content, total polyphenol content, and antioxidant properties (13). The miniaturization of food particles leads to an increase in surface area and porosity and unique physical and chemical properties, such as good solubility, dispersibility, adsorption, biological activity, chemical activity, etc. The application of mulberry leaf powder in the food industry can not only reuse waste raw materials but also improve the health value of food.

Bread is a popular baked food whose main constituent is carbohydrates. Bread made from refined flour has a low fiber, protein, and active phenolic component content. Some natural ingredients have been applied in bread processing to improve the quality and functional properties. It was reported that the addition of spinach powder, green tea powder, and mallow powder improved the content of fiber, protein, and antioxidant properties of the bread (14–16). It was comprehensively reported that mulberry leaf was medicinal and edible; however, food products containing mulberry leaf powder are relatively rare in the market. In this study, the functional components of mulberry leaves were further improved by GABA enrichment, adding enriched GABA mulberry leaf powder to improve the health value of bread. It is a significant step in promoting mulberry leaf powder as a functional food ingredient.

## Materials and Methods

### Reagents and Chemicals

The mulberry leaves (“Yu711”) were collected from the National Mulberry Germplasm Resources in Zhenjiang, Jiangsu Province between March and April. γ-Aminobutyric acid (≥99%) was purchased from Sigma-Aldrich Co., Ltd. (St. Louis, MO, United States). Neochlorogenic acid, chlorogenic acid, cryptochlorogenic acid, catechin, vanillic acid, caffeic acid, syringic acid, rutin, hyperoside, and aminoguanidine hydrochloride (AG) were purchased from the Shanghai Yuanye Bio-Technology Co., Ltd. (Shanghai, China). 2,2-Diphenyl-1-picrylhydrazyl (DPPH) was purchased from TCI (Shanghai) Chemical Industry Development Co., Ltd. (Shanghai, China). Other reagent solutions were all purchased from Sinopharm Chemical Reagent Co., Ltd. (Shanghai, China).

### Plant Material and Experimental Design

Mulberry leaves were manually picked and transported to the laboratory immediately before use. The leaves were treated by different methods according to Li et al. and Tu et al. with slight modifications (17, 18). They were evenly separated among different treatments as follows: (1) control (CK); (2) sodium glutamate immersion (SG): fresh mulberry leaves soaked in 6.22 g/L sodium glutamate solution for 10 min at 25°C; (3) cold shock with sodium glutamate immersion (CS-SG): fresh mulberry leaves put in a low-temperature environment (0°C) for 10 min and soaked in 6.22 g/L sodium glutamate solution for 10 min at 25°C; (4) sodium glutamate immersion with anoxic (SG-A):fresh mulberry leaves soaked in 6.22 g/L sodium glutamate solution for 10 min at 25°C and anoxic treatment for 8 h at 25°C; (5) repeat method (4) twice (SG-A-T); (6) cold shock with anoxic (CS-A): fresh mulberry leaves put in a low-temperature environment (0°C) for 10 min, and anoxic treatment for 8 h at 25°C; (7) repeat method (6) twice (CS-A-T); (8) cold shock with sodium glutamate immersion with anoxic (CS-SG-A): fresh mulberry leaves put in a low-temperature environment (0°C) for 10 min, soaked in 6.22 g/L sodium glutamate solution for 10 min at 25°C, and anoxic treatment for 8 h at 25°C; (9) repeat method (8) twice (CS-SG-A-T). Afterward, the mulberry leaves were removed and rinsed with distilled water. The treated mulberry leaves were separated into two groups. One was dried in a microwave oven and sieved through a 100-mesh after crushing and then stored for use. Another was ground in liquid nitrogen and stored at −80°C until use.

### γ-Aminobutyric Acid-Enriched Mulberry Leaf Water Extracts Preparation and Component Analysis

According to the methods of Huang et al. (19), 1.0 g of mulberry leaf powder was soaked in 40 ml of distilled water (100°C) and placed in a water bath for 2 h at 40°C. The supernatant was filtered with 0.45-μm films for content determination, and the remaining supernatant was freeze-dried and stored at −20°C.

### Determination of γ-Aminobutyric Acid Content

According to the methods of Tang et al. (20), 1.0 ml of enriched mulberry leaf water extracts (GMLE) was added to 1.0 ml of 0.1 moll sodium tetraborate, 1.2 ml of 6% redistilled phenol solution, and 0.6 ml of 7% sodium hypochlorite solution, heated in a boiling water bath for 10 min and immediately placed in an ice bath for 5 min. The absorbance at 360 nm was determined. GABA served as the reference standard.

### Determination of Total Phenolic Content

The total phenolic content was determined according to the method of Balasubramaniam et al. with a slight modification (21). A total of 1 ml of GMLE was mixed with 1 ml of Folin–Ciocalteu reagent and 2 ml of 10% Na_2_CO_3_ solution, mixed thoroughly, and made up to 10 ml with ddH_2_O. The absorbance was measured at 760 nm after 1 h incubation in a water bath at 40°C. Gallic acid was used as the reference standard.

### Phenolic Components Analysis of Enriched Mulberry Leaf Water Extracts

The phenolic compounds were identified and quantified by reverse-phase HPLC using a C18 column (5 μm, 250 mm × 4.6 mm) on an Agilent 1260 HPLC system equipped with VWD at 280 nm (Agilent Technologies, United States). Mobile phase A was HPLC-grade water containing 4% acetic acid and mobile phase B was acetonitrile. The gradient program was reported previously (18).

### Preparation of GABA-Enriched Mulberry Leaf Powder

The GABA-enriched mulberry leaf powder (GMLP) was prepared by a portable high-speed crusher, and the powders passing through 40–60, 80–100, 120–140, and 200–240 mesh screens, respectively, were obtained using different crushing times. Another portion of the mulberry leaves was crushed and then ground into an ultrafine powder with a QM-3SP04 planetary ball mill, which was passed through a 300–400 mesh. The leaves were prepared into powders of five different particle sizes marked M60, M100, M140, M240, and M400. A total of 100 g of the samples were collected and stored at −20°C.

### Particle Size Distribution and Morphological Observation

#### Particle Size Distribution

The particle size distribution of different GMLP was determined by a laser particle size distribution analyzer and characterized by median diameter (D_50_), specific surface area (A_*sf*_), and particle size width span ([(*D90*−*D10*)/(*D50*)]), where the D_10_, D_50_, D_90_ values represent 10, 50, and 90% cumulative percentiles of total particles.

#### Optical Observations

The powders were observed using an optical microscope (RE-2000A, Shanghai Optical Instrument Co., Ltd., China). A total of 0.05 g of powders of different particle sizes were mixed with dilute glycerol (0.5 mL) on a glass slide and the cell structure was observed under a microscope (× 40). Using image analysis software (Image-Pro Plus 6.0, Media Cybernetics, United States), the roundness, aspect ratio, and box X/Y were calculated (22).

#### Scanning Electron Microscopy

Morphological characterization of the GMLP particles was investigated using a JSM-7500F scanning electron microscopy (SEM) (JEOL Ltd., Japan) operated at an accelerating voltage of 5 kV and a working distance of 10–15 mm. The samples were coated with a 10 nm thick platinum layer to make them conductive (23).

#### Fourier Transform Infrared Spectroscopy

The GMLP of different particle sizes was analyzed by fourier transform infrared spectroscopy (FTIR) spectroscopy analysis (NICOLET-IS10 Spectrometer, Thermo Fisher Scientific, United States). Powder samples were prepared using the KBr pellet method. Spectra were recorded in the range of 4000 to 400 cm^–1^.

### Physicochemical Properties of GABA-Enriched Mulberry Leaf Powder With Different Particle Sizes

#### Water and Oil Holding Capacity of GABA-Enriched Mulberry Leaf Powder

Water holding capacity (WHC) and oil holding capacity (OHC) were measured according to the method of Protonotariou et al. with minor modifications (22). A total of 3.0 g of GMLP (m_1_) was added to water (10 ml) for WHC and oil (10 mL) for OHC in the tube and then centrifuged at 1,500 r/min for 5 min. The supernatant was removed and the tubes were weighed (m_2_). WHC and OHC were calculated as follows:


$$\mathrm{WHC}\,\left(\frac{\text{g}}{\text{g}}\right)=\frac{\left(m_{2}-m_{1}\right)}{m_{1}}$$

$$\mathrm{OHC}\,\left(\frac{\text{g}}{\text{g}}\right)=\frac{\left(m_{2}-m_{1}\right)}{m_{1}}$$

#### Swelling Power of GABA-Enriched Mulberry Leaf Powder

According to the method of Zhang et al. (24), swelling power (SP) was determined with slight modification. A total of 1 g of GMLP was placed in a tube (V_1_) and 10 g of distilled water was added. Then the tube was mixed and incubated in a water bath at 25°C for 12 h to allow the complete swelling of the powder (V_2_). SP was calculated with the following formula:


$$\mathrm{SP}\,\left(\mathrm{mL}/\mathrm{mL}\right)=\frac{V_{2}}{V_{1}}$$

#### Angle of Repose Measurement of GABA-Enriched Mulberry Leaf Powder

The angle of repose was measured according to the method of Zhang et al. with minor modifications (24). First, a funnel was fixed vertically above a piece of paper. Then the powder was poured continuously along the edge of the funnel until the powder cone was formed. The angle of repose was determined by the ratio of the height (h) of the cone to the radius (R) of the bottom of the cone. The angle of repose (θ) was calculated with the following formula:


$$\theta =\tan^{-1}\,2\,h/R$$

#### Dissolution Effect of γ-Aminobutyric Acid and Total Phenolics of GABA-Enriched Mulberry Leaf Powder

The extracts were obtained according to the traditional tea culture method. A total of 1.0 g of GMLP was mixed in 40 mL of distilled water (100 °C) and placed in a water bath at 40°C. The mixture was filtered with 0.45 μm films after 5, 10, 20, 30 40, 50, 60, 80, and 110 min, and the content of GABA and total phenolic (TPL) was measured according to the method in section GABA-enriched mulberry leaf water extracts (GMLE) preparation and component analysis.

### 2,2-Diphenyl-1-Picrylhydrazyl Scavenging Activity Assay

The DPPH radical scavenging activity assay was determined according to He et al. with a slight modification (25). The GMLE lyophilized powder was made up to 0.1, 0.15, 0.2, 0.25, and 0.3 mg/ml with distilled water. A total of 1 ml of GMLE lyophilized powder solution or 1 mg GMLP was added to 1 ml of DPPH radical methanol solution (400 μM) in the dark and the absorbance at 517 nm was measured after 30 min. The DPPH radical scavenging activity (%) was calculated using the formula:


DPPHradicalscavengingactivity(%)

$$=\frac{\mathrm{absorbance}\,\mathrm{of}\,\mathrm{control}-\mathrm{absorbance}\,\mathrm{of}\,\mathrm{sample}}{\mathrm{absorbance}\,\mathrm{of}\,\mathrm{control}}\times 100$$

The half-maximal eliminate concentration (EC_50_) value was calculated from the graph plotted between the percentage of scavenging and the concentration of GMLE.

### Inhibitory Effects of Advanced Glycation End-Products Assays

The procedure of protein and glucose reaction followed that of Szawara-Nowak et al. with minor modifications (26). The GMLE lyophilized powder was made up to 0.2, 0.4, 0.6, 0.8, and 1.0 mg/ml with distilled water. A total of 1 g of BSA and 9 g of glucose were dissolved in 0.1 M phosphate buffer (pH = 7.4) to obtain a control solution containing 10 mg/ml of BSA and 0.5 M of glucose. A total of 1 ml of BSA/glucose test solution was incubated at 55°C for 40 h with or without 1 ml GMLE lyophilized powder solution (or 5 mg GMLP). After incubation, the total fluorescence advanced glycation end-products (AGEs) (Ex380, Em450) of the sample were measured. Triplicate samples were run for each set and the percent inhibition was calculated using the formula:


AGEsinhibitionrate(%)

$$=\frac{\mathrm{absorbance}\,\mathrm{of}\,\mathrm{control}-\mathrm{absorbance}\,\mathrm{of}\,\mathrm{sample}}{\mathrm{absorbance}\,\mathrm{of}\,\mathrm{control}}\times 100$$

The half-maximal inhibition concentration (IC_50_) value was calculated from the graph plotted between the percenta ge of inhibition and the concentration of GMLE.

### Bread Production

Bread dough was made according to the formulations given in Table 1. All the ingredients were mixed in a blender (Donlim DL-T06A, China) for 2 min at 90 rpm and then for 8 min at 150 rpm. The dough was left to stand and ferment for 2 h at 30°C with 80% humidity undercover. The dough was vented for 5 min, rolled, and put in baking molds. Then, the dough was subjected to 30°C for 30 min with 80% humidity undercover. Finally, the dough was baked at 170°C/200°C (top/bottom heat) for 30 min in an oven (Supor K42FK823, China). The bread was left to cool down at room temperature for 2 h for further evaluation.

**TABLE 1:** Ingredients used to make bread.

Ingredients	Control	M60-bread	M100-bread	M140-bread	M240-bread	M400-bread
Flours	180	175.5	175.5	175.5	175.5	175.5
M60 GMLP	/	4.5	/	/	/	/
M100 GMLP	/	/	4.5	/	/	/
M140 GMLP	/	/	/	4.5	/	/
M240 GMLP	/	/	/	/	4.5	/
M400 GMLP	/	/	/	/	/	4.5
Water	108	108	108	108	108	108
Sugar	5.4	5.4	5.4	5.4	5.4	5.4
Salt	1	1	1	1	1	1
Instant yeast	2.1	2.1	2.1	2.1	2.1	2.1
Butter	3.6	3.6	3.6	3.6	3.6	3.6
Milk powder	1.4	1.4	1.4	1.4	1.4	1.4

### Effect of GABA-Enriched Mulberry Leaf Powder on the Physical Properties of Bread

The specific volume of the bread was calculated by the volume/mass ratio using the rapeseed displacement method, expressed in ml/g. L*, a*, and b* color attributes of the crust and crumb were determined with a colorimeter (colorimeter CR-400, Konica Minolta, Japonya). Texture profile analysis (TPA) of bread crumbs was determined by a texture profile analyzer (TMS-PRO, FTS, United States). The bread was cut into 2-cm slices, and TPA was performed using an aluminum 38 mm diameter cylindrical probe and a 100 N load cell. The probe speed, trigger force, degree of compression, and the time-lapse between compression cycles were set to 0.5 mm/s, 0.15 g, 30%, and 5 s, respectively.

The texture properties of the bread were established on the 0th and 3rd day (stored at 4°C) after baking to analyze the degree of staling (27). The degree of aging was calculated using the following equation:


$$B\,S\,d=\frac{H\,3\,d-H\,0\,d}{H\,0\,d}\times 100$$

where BSd is the degree of staling of the bread, H3d is the hardness of the bread slice after 3 days of storage, and H0d is the hardness of the bread slice after 0 days of storage.

### Sensory Evaluation of Bread

An evaluation team was formed by 10 food majors as sensory assessors, and the sensory evaluation of bread was carried out in the same environment. The evaluation standard refers to GB/T 14611-2008 with a slight modification (28), the details are as follows:

(1) Form (20): The shape is complete and full, the lines are clear, and there is no obvious focal spot;
(2) Color (20): the color is uniform, and the edge color is allowed to be darker;
(3) Taste (20): it has a unique fragrance, no peculiar smell, and a good taste;
(4) Tissue (20): the tissue is uniform and fine. The cut surface is spongy and elastic;
(5) Impurities (20): no oil stains, no peculiar smell.

### Statistical Analysis

All experiments were analyzed in triplicate. Results were presented as the mean ± standard deviation (SD). Duncan’s multiple comparison tests were performed to identify the difference between values using SPSS 22.0 (SPSS Inc., Chicago, IL, US). The significant difference was set at *P* < 0.05.

## Results and Discussion

### Effects of Different Treatments on the Contents of γ-Aminobutyric Acid, Total Phenolics, and Phenolic Components in Mulberry Leaves

In this study, the GABA content of mulberry leaves was enriched by methods such as adding exogenous substances, anaerobic treatment, and cold shock. Based on a previous experiment (18), the synergy of enrichment methods was used in this experiment. As shown in Table 2, the GABA content of mulberry leaves in the control was 1.17 mg/g. Short-term sodium glutamate soaking had no significant effect on GABA content, but combined with anaerobic and cold shock treatments, GABA content was significantly increased. The GABA content in the repeat cold shock-sodium glutamate immersion-anoxic twice was the highest, reaching 3.58 mg/g. It was followed by cold shock-sodium glutamate immersion-anoxic, whose GABA content reached 3.00 mg/g. After comparing the effects of the above treatments, one addition cycle treatment was found to affect a significant improvement in GABA content. As the post-harvest time of mulberry leaves increases, the edible quality of mulberry leaves will decline, and more circulation of treatment is not necessary. Compared with sodium glutamate immersion, the GABA content of cold shock-sodium glutamate immersion, sodium glutamate immersion-anoxic, repeat sodium glutamate immersion-anoxic twice, cold shock-anoxic, and repeat cold shock-anoxic twice was significantly increased, where the content was 1.55, 1.65, 1.73, 1.55, and 1.57 mg/g, respectively. In addition, the GABA content of repeat cold shock-anoxic twice and repeat cold shock-sodium glutamate immersion-anoxic twice varied greatly. The results show that compared with control, the GABA content of sodium glutamate immersion was not significantly increased, but there was a significant increase in GABA content for old shock-sodium glutamate immersion, cold shock-sodium glutamate immersion-anoxic, repeat cold shock-sodium glutamate immersion-anoxic twice, and repeat sodium glutamate immersion-anoxic twice. So, the anaerobic treatment and cold shock seem to contribute to the improvement of GABA content. Additionally, adding sodium glutamate soaking during anaerobic and can shock treatment could significantly increase the GABA content. It was noted that sodium glutamate, as a substrate for GABA synthesis, played a key role in increasing the GABA content of mulberry leaves under stress.

**TABLE 2:** Effects of different treatments on the content of active compounds in mulberry leaves.

Methods	GABA (mg/g)	Neochlorogenic acid (mg/g)	Chlorogenic acid (mg/g)	Catechin (mg/g)	Cryptochlorogenic acid (mg/g)	Vanillic acid (mg/g)	Caffeic acid (mg/g)	Syringic acid (mg/g)	Rutin (mg/g)	Hyperoside (mg/g)	Total phenolics (mg GAE/g)
CK	1.17 ± 0.07*^d^*	1.44 ± 0.07*^e^*	23.24 ± 1.16*^e^*	0.46 ± 0.02*^c^*	09.04 ± 0.45*^d^*	0.06 ± 0.003*^b^*	0.22 ± 0.01*^d^*	0.21 ± 0.01*^d^*	3.84 ± 0.19*^e^*	1.46 ± 0.07*^e^*	5.92 ± 0.07*^b^*
SG	1.18 ± 0.08*^d^*	0.54 ± 0.03*^f^*	29.19 ± 0.46*^a^*	0.48 ± 0.02*^c^*	08.85 ± 0.24*^d^*	0.05 ± 0.002*^c^*	0.23 ± 0.01	0.29 ± 0.01*^b^*	4.78 ± 0.24c	1.89 ± 0.10*^cd^*	6.78 ± 0.08*^a^*
CS-SG	1.56 ± 0.06*^c^*	1.72 ± 0.09*^d^*	27.23 ± 1.36*^b^*	0.48 ± 0.02*^c^*	11.86 ± 0.40*^ab^*	0.06 ± 0.003*^b^*	0.28 ± 0.01*^b^*	0.29 ± 0.01*^b^*	5.36 ± 0.27*^b^*	2.35 ± 0.12*^a^*	7.02 ± 0.09*^a^*
SG-A	1.65 ± 0.14*^c^*	2.05 ± 0.10*^c^*	22.16 ± 0.51*^e^*	0.47 ± 0.02*^c^*	11.41 ± 0.16*^bc^*	0.05 ± 0.003*^c^*	0.26 ± 0.01*^c^*	0.21 ± 0.01*^d^*	4.64 ± 0.23*^cd^*	1.77 ± 0.09*^d^*	6.01 ± 0.13*^b^*
SG-A-T	1.73 ± 0.31*^c^*	2.12 ± 0.11*^bc^*	23.48 ± 1.17*^de^*	0.58 ± 0.03*^b^*	12.08 ± 0.41*^ab^*	0.04 ± 0.002*^d^*	0.25 ± 0.01*^c^*	0.15 ± 0.01*^f^*	4.22 ± 0.11*^de^*	1.92 ± 0.01*^cd^*	6.09 ± 0.08*^b^*
CS-A	1.55 ± 0.06*^c^*	2.24 ± 0.10*^ab^*	17.58 ± 0.88*^f^*	0.44 ± 0.02*^c^*	10.98 ± 0.46*^c^*	0.06 ± 0.002*^b^*	0.29 ± 0.01*^b^*	0.17 ± 0.01*^e^*	3.80 ± 0.19*^e^*	1.47 ± 0.07*^e^*	7.06 ± 0.12*^a^*
CS-A-T	1.57 ± 0.07*^c^*	1.38 ± 0.07*^e^*	22.29 ± 1.11*^e^*	0.57 ± 0.03*^b^*	09.51 ± 0.57*^d^*	0.06 ± 0.003*^b^*	0.23 ± 0.01*^d^*	0.20 ± 0.01*^d^*	4.48 ± 0.22*^cd^*	1.93 ± 0.10*^c^*	5.99 ± 0.61*^b^*
CS-SG-A	3.0 ± 0.12*^b^*	2.13 ± 0.11*^bc^*	25.00 ± 1.20*^cd^*	0.45 ± 0.02*^c^*	12.42 ± 0.13*^a^*	0.05 ± 0.002*^c^*	0.33 ± 0.01*^a^*	0.27 ± 0.01*^c^*	5.44 ± 0.27*^b^*	2.10 ± 0.11*^b^*	6.21 ± 0.18*^b^*
CS-SG-A-T	3.58 ± 0.15*^a^*	2.38 ± 0.10*^a^*	26.08 ± 1.03*^bc^*	1.25 ± 0.04*^a^*	11.47 ± 0.38*^bc^*	0.09 ± 0.004*^a^*	0.29 ± 0.01*^b^*	0.35 ± 0.02*^a^*	6.65 ± 0.33*^a^*	2.12 ± 0.11*^b^*	6.34 ± 0.31*^b^*

*Results were represented as mean ± SD (n = 3); a, b, c, d, e, f means in the same column with common superscripts are not significantly different whereas values with different superscripts are significantly different at p < 0.05.*

*CK, control; SG, sodium glutamate immersion; CS, cold shock; A, anoxic; T, repeat twice.*

Phenolics are bioactive compounds in mulberry leaves, which have strong antioxidant properties and can prevent cardiovascular and cerebrovascular diseases (29). Mencin et al. reported that exogenous factors, such as salinity, temperature, and mechanical damage, affected phenolic biosynthesis in germinated spelled wheat seeds (10). Tu et al. found that phenolic profiles in mulberry leaves were changed under sodium glutamate immersion (18). It was shown that GABA accumulation was closely related to the secondary metabolism of mulberry leaves, and brings potential biological activities. As shown in Table 2, compared with the control (5.92 mg/g), the content of total phenolics was significantly increased by sodium glutamate immersion, cold shock-sodium glutamate immersion, and cold shock-anoxic, which were 6.78, 7.02, and 7.06 mg/g, respectively, while other methods had no significant effect on total phenolic content. Furthermore, it is important to note that, although there was no significant difference in total phenol content during GABA enrichment, some active phenolic components were significantly increased. Compared with the control, repeat cold shock-sodium glutamate immersion-anoxic twice treatment significantly increased the contents of some active phenolic components, including neochlorogenic acid, chlorogenic acid, catechin, cryptochlorogenic acid, vanillic acid, caffeic acid, syringic acid, rutin, hypericin, which increased by 65.28, 12.22, 171.73, 27.10, 50.00, 31.82, 66.67, 73.18, and 45.21%, respectively. It has been reported that these compounds had antioxidant, hypoglycemic, antibacterial, and antitumor activities (30). Under stress, plants were stimulated to produce phenolic components by inducing endogenous hormones to resist adverse environments (31). In this study, the combined method avoided long-term soaking and effectively enriched the active GABA and phenolic components of mulberry leaves.

### Effects of Cold Shock With Sodium Glutamate Immersion With Anoxic Repeat Twice Treatment on 2,2-Diphenyl-1-Picrylhydrazyl and Advanced Glycation End-Products Inhibitory Activity

**FIGURE 1:**
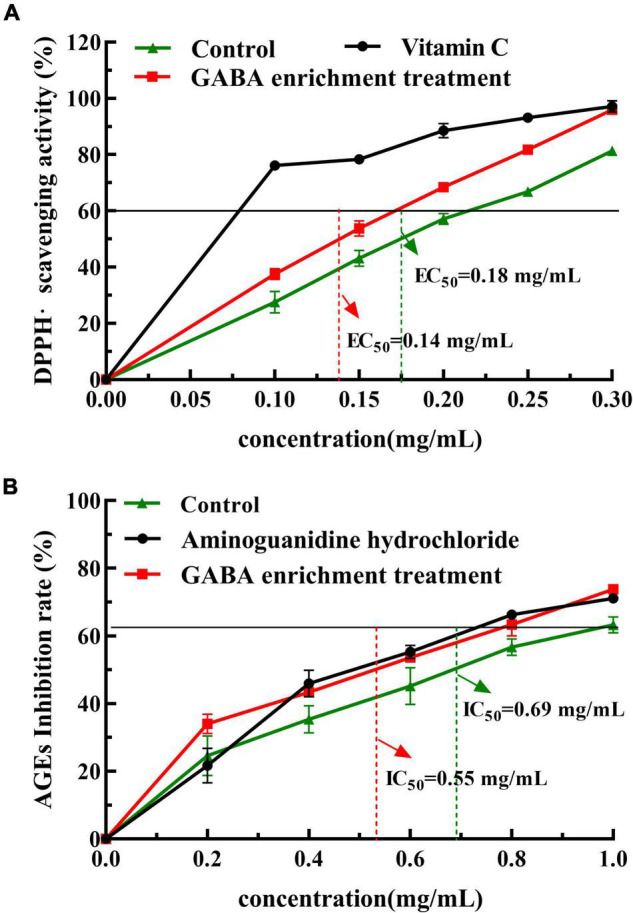
Antioxidant and AGEs inhibitory activities of extracts from GABA-enriched mulberry leaf [note: GABA enrichment treatment: cold shock-sodium glutamate immersion- anoxic, repeat twice; The extract from untreated mulberry leaf as a control, Vitamin C as a positive control in **(A)**, aminoguanidine hydrochloride as a positive control in **(B)**].

It is reported that GABA, polyphenols, and flavonoids in mulberry leaves have anti-oxidant, hypoglycemic, and other effects (32). The repeat cold shock–sodium glutamate immersion–anoxic twice treated group significantly increased GABA and phenolic content (as shown in Table 2), requiring the determination of the inhibitory activities of DPPH radical and AGEs to evaluate the effect of GABA enrichment on the health value of mulberry leaves. Figure 1A shows that the DPPH radical scavenge capacity was gradually enhanced with the increase in the concentration of the water extract of mulberry leaves, where the EC_50_ value of DPPH radical scavenge in the repeat cold shock-sodium glutamate immersion-anoxic twice group was 0.14 mg/ml.

Advanced glycation end-products are structurally complex compounds formed by carbonyl and amino groups at the end of the Maillard reaction. Its excessive accumulation in organs, tissues, and the circulatory system aggravates the body’s oxidative stress state and inflammatory response, causing diseases such as diabetes, atherosclerosis, and Alzheimer’s disease (33). Figure 1B shows that the inhibitory effect on the production of AGEs was gradually enhanced with the increase of the concentration of the mulberry leaves water extract (0–1.0 mg/mL). The IC_50_ value of AGEs in the repeat cold shock-sodium glutamate immersion-anoxic twice group was 0.55 mg/mL, which was significantly decreased (the control group was 0.69 mg/mL). The results indicate that GABA-enriched mulberry leaves have a strong inhibitory capacity on protein glycosylation in the thermal processing mode of food.

### Particle Size Analysis and Particle Description of GABA-Enriched Mulberry Leaf Powder

Crushing is a common method of processing raw materials for food to improve both processing characteristics and the nutritional value. Superfine grinding has become increasingly used recently. In this study, GMLP with different particle sizes was prepared. The D_50_, A_*sf*,_ and span of different sized particulate GMLP are shown in Table 3. The D_50_ values of GMLPs decreased from 404.0 to 18.6 μm, which indicates that the M400 sample belonged to the superfine food power grade (11). The A_*sf*_ value increased from 12.9 to 246.8 m^2^/kg with a decreasing particle size. Surface energy and the active sites increased with decreasing particle size, which led to an increase in the contact area between the powder and the solvent, and was conducive to the dissolution of intracellular components. The span value is usually used to measure particle size distribution. The span values of superfine powders (M400) were significantly larger than coarse granulates (M60, M100, M140, and M240), indicating that the M400 sample had a wider particle size distribution.

**TABLE 3:** Particle size distributions of the GABA-enriched mulberry leaf powder (GMLP).

Sample	D_10_ (μ m)	D_50_ (μ m)	D_90_ (μ m)	A_*sf*_ (m^2^/kg)	Span
M60	223.2 ± 2.3*^a^*	404.0 ± 3.8*^a^*	621.8 ± 4.8*^a^*	012.9 ± 0.7*^e^*	0.986 ± 0.003*^c^*
M100	126.2 ± 1.1*^b^*	224.9 ± 2.7*^b^*	344.2 ± 2.4*^b^*	019.6 ± 1.1*^d^*	0.969 ± 0.010*^c^*
M140	069.7 ± 0.6*^c^*	106.0 ± 1.0*^c^*	151.6 ± 0.8*^c^*	029.7 ± 1.4*^c^*	0.871 ± 0.003*^d^*
M240	009.1 ± 0.1*^d^*	069.3 ± 0.5*^d^*	112.0 ± 0.4*^d^*	097.5 ± 2.9*^b^*	1.485 ± 0.010*^b^*
M400	003.9 ± 0.1*^e^*	018.6 ± 0.1*^e^*	061.9 ± 0.5*^e^*	246.8 ± 3.5*^a^*	3.126 ± 0.044*^a^*

*Results were represented as mean ± SD (n = 3); a, b, c, d, e means in the same column with common superscripts are not significantly different whereas values with different superscripts are significantly different at p < 0.05.*

### Morphology of GABA-Enriched Mulberry Leaf Powder With Different Sizes

**FIGURE 2:**
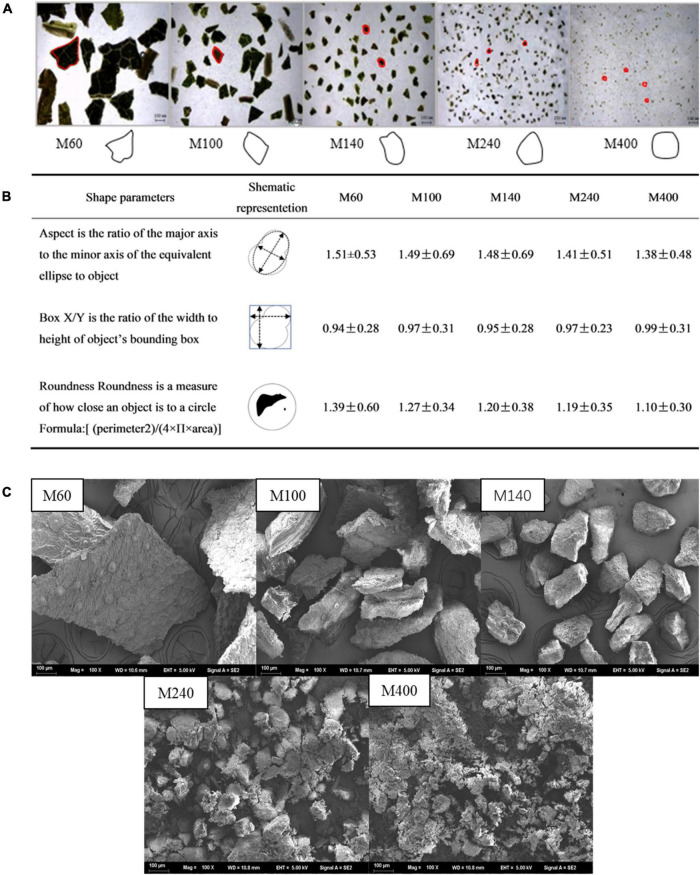
Morphology observation of the GABA-enriched mulberry leaf powder (GMLP). **(A)** Microscope images with indicant mean diameters of different powder fractions M60, M100, M140, M240, M400, and schematic simulation of granules; **(B)** Shape factors definition and values of different mulberry leaves powder fractions; **(C)** SEM images of different mulberry leaf powder sizes.

Microscopic images were obtained for five sizes of GMLP, and the results are shown in Figure 2A, where fragments of different particle shapes coexist in the mulberry leaf powder at different mesh sizes. The cell morphology of M100 GMLP was seen with a complete cell wall, but the cell wall was difficult to see if the particle size was within the range of the 120–240 mesh (M240) and only some of the cell debris was seen in M400. Based on the data obtained from microscopic morphology, the most common particle shape groups in different samples were graphically simulated. The quantitative data on these shape factors are shown in Figure 2B. As the particle size decreased, the shape changed significantly. The Box X/Y value of the smallest particle size particle (M400) was 0.99 and the shape was closer to a circle, while the largest particle(M60) was a more polygonal shape. Furthermore, M400 had the most uniform shape with regular symmetry followed by the M240 and M100 samples. M60 and M140 samples with lower BoxX/Y values showed irregular shapes. SEM images were also obtained in Figure 2C. The results showed that the fiber structures of the particles were gradually destroyed as the particle size decreased. The fibers of the M400 sample were observed to become smaller, and most of the cell structure was destroyed, which was consistent with the microscopic results. These results indicate that GMLP with different particle sizes has different processing properties.

### Fourier Transform Infrared Spectroscopy of GABA-Enriched Mulberry Leaf Powder With Different Sizes

**FIGURE 3:**
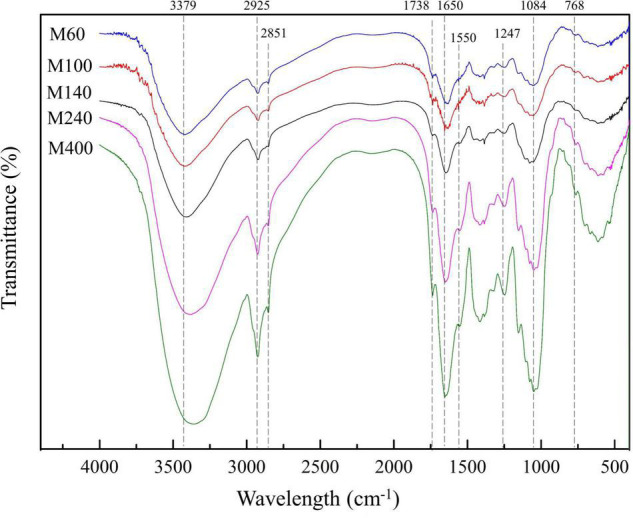
FTIR spectra of the GABA-enriched mulberry leaf powder (GMLP).

Figure 3 shows the FTIR results of GMLP, which helped to distinguish the changes in the key bioactive components composition of GMLP. In the FTIR spectra, peaks at around 3379, 2925, 2851, 1738, 1650, 1550, 1247, 1084, and 768 cm^–1^ were observed. The spectral characteristics of GMLP with different particle sizes were similar, and there were no new chemical group bands produced as the particle size decreased. The band at around 3379 cm^–1^ is associated with the stretching vibration band of the intermolecular hydrogen bond O-H. Compared with the FTIR spectrum of the GMLP of M60, the characteristic absorption bands of M100, M140, M240, and M400 at 3,379cm^–1^ shifted to a lower wavenumber around 3,306 cm^–1^, which indicates that the mechanical force during superine grinding processing could break the intramolecular hydrogen bonds (34). The band at around 3,322 cm^–1^ corresponds to the –OH stretch vibration and the band at 1.741 cm^–1^ is attributed to the stretching band of the carbonyl (*C* = O) group in the phenolic structure of grape pomace powder (35). In the present study, bands around 3,306 cm^–1^ and 1,738 cm^–1^ indicate the presence of phenolic substances in GMLP. The wavelengths at these absorption bands did not change, indicating that the main structure of phenolic molecules was not destroyed during superfine grinding (34). The bands located at 2,925 cm^–1^ and 2,851 cm^–1^, respectively, represented C-H asymmetric stretching and symmetric stretching vibrations in alkanes (36). Those occurring at 1,650 cm^–1^ and 1,550 cm^–1^ correspond to the *C* = O group stretching vibrations and the N-H deformation vibrations of the protein, respectively (37). The band at 1,084 cm^–1^ is the stretching vibration peak formed by the C-O-H in the polysaccharide and the C-O-C in the pyranose ring. The band at 768 cm^–1^ was considered to be an aromatic ring (38). It was found that the smaller the particle size of the GMLP, the stronger the band strength. This might be related to the increase in external surface area and porosity associated with the decrease in particle size. The smaller the particle size, the higher the absorbance and the narrower the band because the particles behave as thin incoherent scatterers (39). These results imply that the exposure of the chemical functional groups of the GMLP are directly related to the particle size, which is due to the changes in the surface of the powder during the grinding process. This is consistent with the results from electron microscopy (Figure 2C).

### Physicochemical Properties of GABA-Enriched Mulberry Leaf Powder

Water holding capacity (WHC), swelling capacity (SP), and oil holding capacity (OHC) are common physicochemical properties of powdered raw materials. The WHC, OHC, and SP values of GMLP with different particle sizes were determined. As shown in Table 4, the WHC, OHC, and SP values of GMLP were reduced when the particle sizes were reduced. M400 GMLP had the lowest values. Similar observations were reported in citrus pomace and mushroom powders (24, 40). The surface area of the superfine powder was much larger than the coarse powder, and the contact area with water was larger, while the above values were affected by the cell water holding tissues which were severely damaged by the mechanical force. However, some studies have found that WHC and SP values increase with the decrease in particle size, such as in *Dendrobium officinale* powder (23). The results imply that the hydration properties of the material are not only affected by the particle size, but also by the composition, surface properties, hydrophobicity, and especially the type of material. The grinding process usually had different effects on the powders. On the one hand, grinding could increase the porosity of the fiber to a certain extent and the hydration site could increase the WHC and SP values (41). On the other hand, grinding destroyed cell structure and the original larger fiber network structure, which weakened the water absorption effect and the binding force of the powder with water molecules. The hydration of the micro-powder was reduced when the latter effect was dominant (40). The OHC decreased with the decrease in particle size. It was inferred that the specific surface area and surface activity were significantly increased. Some particles were agglomerated, which reduced the contact area with the grease, resulting in a decrease in the OHC.

**TABLE 4:** Physicochemical properties of the GABA-enriched mulberry leaf powder (GMLP).

Sample	Water holding capacity (g/g)	Oil holding capacity (g/g)	Swelling power (mL/mL)	Angle of repose (°)	DPPH radical⋅ scavenging capacity (%)	Inhibition rate of AGEs (%)
M60	5.61 ± 0.12*^d^*	2.02 ± 0.02*^e^*	4.10 ± 0.10*^e^*	35.63 ± 0.83*^a^*	13.48 ± 1.99*^a^*	31.76 ± 5.22*^a^*
M100	4.68 ± 0.03*^c^*	1.73 ± 0.02*^d^*	3.67 ± 0.06*^d^*	45.25 ± 1.95*^b^*	23.40 ± 2.95*^b^*	52.57 ± 5.83*^c^*
M140	4.33 ± 0.11*^c^*	1.62 ± 0.09*^c^*	3.30 ± 0.10*^c^*	49.00 ± 1.86*^c^*	31.52 ± 3.53*^c^*	67.21 ± 5.25*^d^*
M240	3.80 ± 0.09*^b^*	1.49 ± 0.06*^b^*	2.87 ± 0.06*^b^*	52.18 ± 0.46*^d^*	48.43 ± 3.06*^d^*	44.14 ± 7.26*^b^*
M400	3.51 ± 0.04*^a^*	1.38 ± 0.01*^a^*	2.50 ± 0.10*^a^*	53.48 ± 1.06*^e^*	65.01 ± 2.44*^e^*	41.49 ± 5.12*^ab^*

*Results were represented as mean ± SD (n = 3); a, b, c, d, e means in the same column with common superscripts are not significantly different whereas values with different superscripts are significantly different at p < 0.05.*

### Powder Fluidity Analysis

The angle of repose is an intuitive indicator reflecting the change of powder fluidity. The larger the angle of repose, the worse the fluidity of the powder. Table 4 indicated that the angle of repose of the sample increased from 35.63° to 53.48° as the particle size of the powder decreased, which indicates that M400 has worse fluidity. The powder agglomeration force increased with the decrease of the particle size, which made the particles stick together closely and formed a steeper angle (35).

### Dissolution Rate of γ-Aminobutyric Acid and Total Phenolic in GABA-Enriched Mulberry Leaf Powder

**FIGURE 4:**
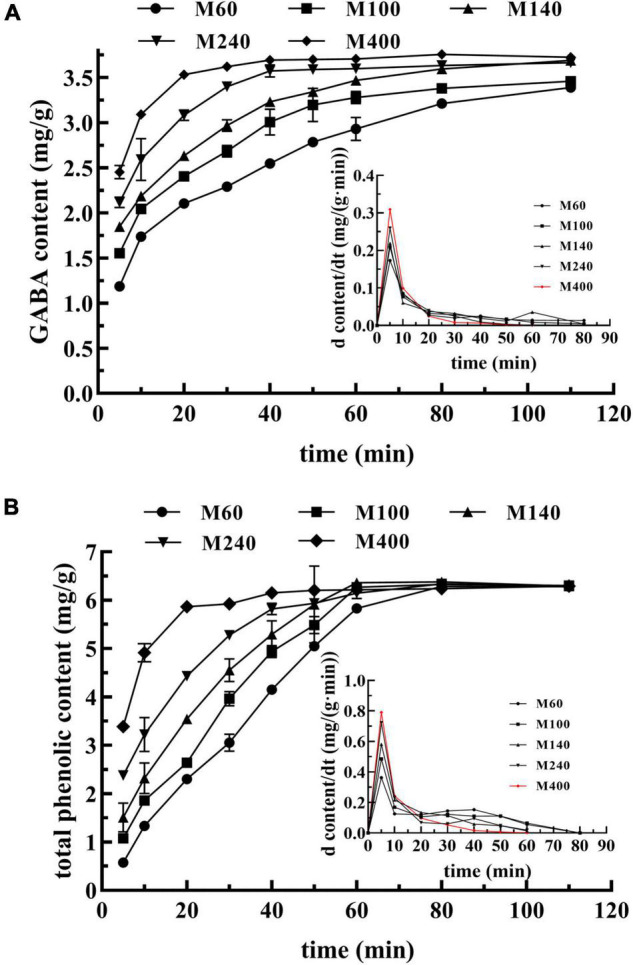
Dissolution of different components from the GABA-enriched mulberry leaf powder (GMLP). **(A)** GABA, **(B)** TPL.

The chemical composition dissolution of different granulometric powders was determined. The content of GABA and TPL in the water extract at different extraction times is shown in Figure 4. The results show that GABA and TPL had a high diffusion rate in the initial stage and gradually leveled off. In Figure 4A, the dissolution rate and content of GABA in GMLP were improved by reducing the particle size. The dissolution of GABA in the M400 sample was up to 3.10 mg/g at 10 min, while that of the M60 sample only reached 1.73 mg/g. Similar results were observed in the dissolution of TPL. It is suggested that particles with a high specific surface area contact water sufficiently during the extracting process to enhance dispersibility and reduce the diffusion time of solute through the particle-matrix (42). Meanwhile, as the particle size decreased, the equilibrium dissolution of GABA increased, but the dissolution of TPL was not significantly different between particle sizes (Figure 4B), which may be related to the oxidation of the dissolved phenolic compounds.

### Effects of GABA-Enriched Mulberry Leaf Powder on 2,2-Diphenyl-1-Picrylhydrazyl Scavenging Activity

2,2-Diphenyl-1-picrylhydrazyl radical scavenging capacity was investigated to value the antioxidant capacity of different mesh sizes of mulberry leaf powder. In Table 4, the DPPH radical scavenging activity is shown to gradually increase with the increase of the powder’s mesh number. With the M400 sample, the scavenging rate reached 65.01%, indicating that the smaller the particle size of the mulberry leaf powder, the greater the DPPH radical scavenging ability. Meng et al. also reported similar results in *Dendrobium officinale* powder (23). It is speculated that the reduction in the particle size of mulberry leaves may lead to faster release and more effective substances to bind free radicals.

### Inhibitory Effects of GABA-Enriched Mulberry Leaf Powder on Advanced Glycation End-Products

Normally, the thermal processing of food produces a variety of AGEs, and the food-borne AGEs are the main source of AGEs in the human body (43). In this study, the BSA/glucose system was used to evaluate the inhibitory effect of GABA-enriched mulberry leaf powder on the formation of AGEs. It can be seen from Table 4 that the inhibition rate of AGEs first increased and then decreased with the increase of the mesh of mulberry leaf powder. M140 has the strongest inhibition rate on AGEs production, where the inhibition rate reached 67.21% when 5 mg GMLP was added to the BSA/glucose system. This shows that different mesh sizes of mulberry leaf powder have different inhibitory effects on AGEs. The inhibitory activities may be related to the dissolution pattern of the antioxidant components in the powder, and the M100 and M140 samples get the right balance between the dissolution of phenolic components and playing inhibitory roles.

### Effects of GABA-Enriched Mulberry Leaf Powder on the Quality of Bread

The physical properties of the bread on adding GMLP of different particle sizes were determined, and the results are shown in Table 5. The specific volume of bread is an important parameter for evaluating visual quality. Compared with the control, the specific volume of bread decreased with the addition of mulberry leaf powder (*p* < 0.05). Junejo et al. reported that adding spinach powder reduced the specific volume of bread (14). Mulberry leaves contain a large amount of dietary fiber, which causes the uniformity and continuity of the gluten network to be interrupted, thereby reducing the gas retention in the bread structure. Compared with the control, the hardness value of the sample with M60-M240 mulberry leaf powder was not significantly different, but the hardness value of the sample with M400 mulberry leaf powder was significantly increased. Bread staling refers to the decline in quality during storage, resulting in the disappearance of bread flavor, poor taste, and economic waste (44). Therefore, it is of great practical significance to delay the staling of bread and prolong its shelf life. In this study, the use of mulberry leaf powder significantly delayed the staling process of the bread. Adding M140-M400 mulberry leaf powder significantly delayed the degree of staling in the bread, and the M240 powder showed the maximum potential for alleviating staling in the bread. Rossana et al. found similar results when studying the effect of different particle sizes of wheat bran on the quality of bread. As the particle size of wheat bran flour decreased, the bread hardness showed a trend of firstly decreasing and then increasing. When the particle size was 160 μm, the bread crumb showed the lowest hardness (45). However, there are few studies on the effects of different particle size additives on the bread quality, and further studies are needed to display the role of particle size of bioprocessing-induced changes in the nutritional and functional properties of mulberry leaves.

**TABLE 5:** The effects of GMLP with different particle sizes on the quality of bread.

		Control	M60-bread	M100-bread	M140-bread	M240-bread	M400-bread
Hardness(after 2 h)		4.5 ± 0.2*^b^*	4.7 ± 0.1*^b^*	4.4 ± 0.3*^b^*	4.7 ± 0.3*^b^*	4.4 ± 0.1*^b^*	5.5 ± 0.1*^a^*
Degree of staling(%)		391.5 ± 5.8*^a^*	387.8 ± 32.4*^a^*	380.0 ± 3.07*^a^*	267.3 ± 63.5*^b^*	180.6 ± 42.8*^c^*	229.1 ± 69.0*^bc^*
Specific Volume(mL/g)		2.35 ± 0.11*^a^*	2.23 ± 0.12*^a^*	2.23 ± 0.10*^a^*	2.22 ± 0.14*^a^*	2.26 ± 0.12*^a^*	2.20 ± 0.05*^a^*
Color of bread crumb	L*	60.8 ± 5.7*^a^*	56.5 ± 1.3*^b^*	52.8 ± 1.0*^c^*	52.2 ± 2.6*^d^*	50.7 ± 3.1*^e^*	48.0 ± 0.4*^f^*
	a*	1.5 ± 0.2*^a^*	1.3 ± 0.1*^b^*	1.0 ± 0.1*^c^*	0.9 ± 0.2*^d^*	0.8 ± 0.3*^e^*	0.7 ± 0.1*^f^*
	b*	11.6 ± 0.5*^e^*	11.9 ± 0.5*^e^*	15.1 ± 0.9*^d^*	16.2 ± 1.8*^c^*	20.6 ± 1.3*^b^*	23.2 ± 0.8*^a^*
Color of bread crust	L*	72.0 ± 4.0*^a^*	65.9 ± 1.7*^b^*	63.0 ± 3.7*^c^*	60.8 ± 1.2*^d^*	57.1 ± 0.8*^e^*	53.6 ± 2.0*^f^*
	a*	2.8 ± 0.3*^a^*	2.6 ± 0.2*^b^*	1.5 ± 0.2*e*	1.3 ± 0.3*^f^*	2.1 ± 0.2*^c^*	2.0 ± 0.4*d*
	b*	20.5 ± 0.9*^f^*	23.8 ± 2.0*^e^*	22.4 ± 1.0*^d^*	25.2 ± 1.4*^c^*	28.7 ± 1.1*^b^*	34.4 ± 0.6*^a^*
Sensory evaluation of bread	Form	18.1 ± 0.9*^a^*	17.6 ± 0.9*^a^*	16.5 ± 0.4*^bc^*	17.6 ± 0.7*^ab^*	17.6 ± 0.4*^ab^*	17.3 ± 0.4*^ab^*
	Color	18.6 ± 0.9*^a^*	13.2 ± 1.9*^d^*	15.6 ± 0.5*^c^*	16.4 ± 0.9*^bc^*	17.0 ± 0.3*^c^*	17.2 ± 0.5*^c^*
	Taste	16.1 ± 0.6*^c^*	14.3 ± 1.7*^e^*	14.4 ± 0.7*^d^*	16.4 ± 0.6*^ab^*	17.6 ± 0.4*^ab^*	15.2 ± 0.5*^d^*
	Tissue	17.9 ± 0.7*^a^*	16.0 ± 1.6*^d^*	16.1 ± 0.7*^d^*	17.6 ± 0.6*^b^*	16.8 ± 0.8*^bc^*	16.2 ± 0.6*^b^*
	Imputrities	19.2 ± 0.8*^a^*	17.8 ± 0.6*^b^*	18.4 ± 0.5*^ab^*	18.7 ± 0.4*^ab^*	18.5 ± 0.5*^ab^*	19.2 ± 0.6*^a^*
	Total score	89.9 ± 4.0*^a^*	78.9 ± 6.7*^b^*	81.0 ± 2.9*^b^*	86.6 ± 3.3*^a^*	87.6 ± 2.5*^a^*	85.2 ± 2.5*^ab^*

*Results were represented as mean ± SD (n = 3); a, b, c, d, e, f means in the same raw with common superscripts are not significantly different whereas values with different superscripts are significantly different at p < 0.05.*

**FIGURE 5:**
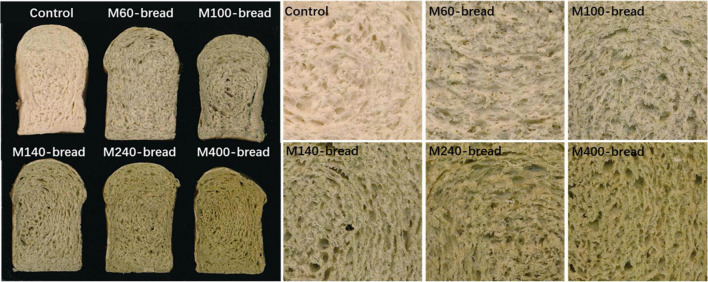
Effect of GMLP on the physical appearance of bread.

The L, a, and b parameters of the crust and crumb color of the bread are shown in Figure 5 and Table 5. Mulberry leaf powder reduced the L* value of bread crumbs and crusts. As the meshes of mulberry leaf powder increased in size, the L* value gradually decreased. The a* value of crumbs and crusts decreased with the addition of mulberry leaf powder, whereas the b* value showed a rising trend. The results show that the addition of mulberry leaf powder darkened the color of the bread and increased the greenness and yellowness. It can be seen in Figure 5 that the mulberry leaf particles were seen in the M60-M100 powder added to the bread, while the M120-M400 mulberry leaf powder was evenly integrated into the flour. The change of bread color may be related to mulberry leaf pigment and particle size.

The sensory evaluation results of mulberry leaf bread are shown in Table 5. The sensory evaluation of bread added with mulberry leaf powder of different particle sizes was different from that of the control group. Since the mulberry leaf powder of M60 and M100 has a larger particle size and a rougher taste, its sensory score was lower. Bread with M140 and M240 mulberry leaf powder has better taste, color, and unique fragrance of mulberry leaf powder. Therefore, the sensory scores of the bread with M140 and M240 mulberry leaf powder are higher, which reach 85.1 and 86.6 points, respectively. The sensory score of bread on adding M400 mulberry leaf powder decreased, which is possibly due to its increased hardness and poor taste. In summary, adding the powders effectively alleviated the staling of bread without any significant effect on taste.

In this study, it is shown that the enrichment of GABA in mulberry leaves increases the contents of active phenolic components, and both the DPPH radical scavenging ability and the AGEs inhibitory capacity were increased. After being prepared into powders with different particle sizes, the mulberry leaves were used for bread baking. When the addition amount was 2.5%, the 140–400 mesh powders significantly alleviated the staling of the bread and had better taste. This indicates that the GABA-rich mulberry leaf powder had a potential function in prolonging the shelf life of certain foods.

## Data Availability Statement

The original contributions presented in the study are included in the article/supplementary material, further inquiries can be directed to the corresponding authors.

## Author Contributions

YJ: partial data collection, methodology, and writing-original draft preparation. JT: majority of data collection and visualization. XH: investigation. JZ: visualization and investigation. GL: partial data collection. YH and HD: writing-reviewing. JW: resources. HX: conceptualization, validation, and supervision. All authors contributed to the article and approved the submitted version.

## Conflict of Interest

The authors declare that the research was conducted in the absence of any commercial or financial relationships that could be construed as a potential conflict of interest.

## Publisher’s Note

All claims expressed in this article are solely those of the authors and do not necessarily represent those of their affiliated organizations, or those of the publisher, the editors and the reviewers. Any product that may be evaluated in this article, or claim that may be made by its manufacturer, is not guaranteed or endorsed by the publisher.
